# The Effects of Antofine on the Morphological and Physiological Characteristics of *Phytophthora capsici*

**DOI:** 10.3390/molecules29091965

**Published:** 2024-04-25

**Authors:** Mei Wang, Weirong Zhang, Jiaojiao Lu, Yanbo Huo, Jing Wang

**Affiliations:** 1 College of Life Science, Yulin University, Yulin 719000, China; zhangwei11rong@163.com (W.Z.); Lj126957865@126.com (J.L.); huoyanbo@163.com (Y.H.); 2 College of Chemistry and Chemical Engineering, Yulin University, Yulin 719000, China; wj2019001@yulinu.edu.cn

**Keywords:** antofine, *Phytophthora capsici*, respiratory chain complexes, biological characterization, mycelia morphology

## Abstract

*Phytophthora capsici* is an important plant pathogenic oomycete that causes great losses to vegetable production around the world. Antofine is an important alkaloid isolated from *Cynanchum komarovii* Al. Iljinski and exhibits significant antifungal activity. In this study, the effect of antofine on the mycelial growth, morphology, and physiological characteristics of *P. capsici* was investigated using colorimetry. Meanwhile, the activity of mitochondrial respiratory chain complexes of *P. capsici* was evaluated following treatment with a 30% effective concentration (EC_30_), as well as EC_50_ and EC_70_, of antofine for 0, 12, 24, and 48 h. The results showed that antofine had a significant inhibitory effect against *P. capsici*, with an EC_50_ of 5.0795 μg/mL. After treatment with antofine at EC_50_ and EC_70_, the mycelia were rough, less full, and had obvious depression; they had an irregular protrusion structure; and they had serious wrinkles. In *P. capsici*, oxalic acid and exopolysaccharide contents decreased significantly, while cell membrane permeability and glycerol content increased when treated with antofine. Reactive oxygen species (ROS) entered a burst state in *P. capsici* after incubation with antofine for 3 h, and fluorescence intensity was 2.43 times higher than that of the control. The activities of the mitochondrial respiratory chain complex II, III, I + III, II + III, V, and citrate synthase in *P. capsici* were significantly inhibited following treatment with antofine (EC_50_ and EC_70_) for 48 h compared to the control. This study revealed that antofine is likely to affect the pathways related to the energy metabolism of *P. capsici* and thus affect the activity of respiratory chain complexes. These results increase our understanding of the action mechanism of antofine against *P. capsici*.

## 1. Introduction

*Phytophthora capsici* is an important plant pathogenic oomycete that often harms the production of many crops, such as those from the Solanaceae and Cucurbitaceae families, and causes great losses to vegetable production around the world [1,2]. The phytophthora blight caused by *P. capsici* can rapidly escalate into a disaster, harming the entire growth cycle of peppers; damaging their seedlings, adult stems, fruits, and leaves; and resulting in a large number of dead seedlings, dead plants, and rotten fruits [3]. Therefore, the phytophthora blight caused by *P. capsici* poses a significant threat to pepper production and can lead to the death of the whole plant or most of the fruit rotting, even resulting in little to no harvest in a year. At present, metalaxyl and mefenoxam are commonly used for the prevention and treatment of phytophthora blight globally; however, these agents have a single site of action, making *P. capsici* prone to developing resistance [4,5]. The resistance of *P. capsici* to metalaxyl has been reported in many studies [4,5,6]. However, biofungicides inhibit pathogens synchronously through several mechanisms that delay resistance development [7]. Hence, a new biofungicide with low toxicity and environmental friendliness is urgently needed to control phytophthora blight.

Alkaloids are a class of nitrogen-containing alkaline organic compounds that exist in nature. Alkaloids are widely distributed in a variety of plants, most of which are dicotyledonous plants [8]. Alkaloids are active components in traditional Chinese medicine and are often used for their anti-inflammatory, antibacterial, antihypertensive, and antitumor properties [9,10,11,12]. According to their different chemical structures, alkaloids are mainly classified into amines, pyridines, pyrrolidines, quinolines, isoquinolines, indoles, tropanes, steroids, and terpenes [8]. Antofine, also known as 7-demethoxytylophine, is an important phenanthroindolizidine alkaloid isolated from *Cynanchum komarovii* Al. Iljinski that belongs to the Asclepiaceae family, *Cynanchum* Linn genus. These plants are widely distributed in the northwest of China, with extreme tolerance to drought and high temperatures [13]. The extraction of antofine has been described previously by Wiegrebe et al. [14]. Antofine shows antifungal and antitumor activities and is associated with low toxicity and no threat to environmental health [15,16,17,18]. In our previous study, we found that antofine possesses significant antifungal activity against *Fusarium semitectum*, *Rhizoctonia solani*, *Setosphaeria turcica*, *Botrytis cinerea*, and *Valsa mali* [18]. In addition, antofine has a significant inhibitory effect on *Sclerospora graminicola* with an EC_50_ of 5.2245 μg/mL. *S. graminicola* is an obligate biotrophic oomycete that causes serious downy mildew disease in foxtail millet. Antofine can effectively prevent and control the incidence of millet downy mildew in field experiments. Considering the antifungal activity of antofine, the inhibitory effect on oomycetes is particularly significant. However, the mechanism of action of antofine is still unclear, warranting further study about its effect against *P. capsici*. Hence, the objective of this study was to determine the effect of antofine on the morphological, physiological, and biochemical characteristics of *P. capsici*.

## 2. Results

### 2.1. Effect of Antofine on the Mycelial Growth of P. capsici

The mycelial growth inhibition rates ranged from 32.61% to 87.22% when treated with antofine at different concentrations (2.5, 5, 10, 20, and 40 μg/mL). The results show the significant inhibitory effect of antofine against *P. capsici* with EC_30_, EC_50_, and EC_70_ values of 1.2891, 5.0795, and 19.0233 μg/mL, respectively (Table 1).

### 2.2. Effect of Antofine on the Mycelial Morphology of P. capsici

**Figure 1:**
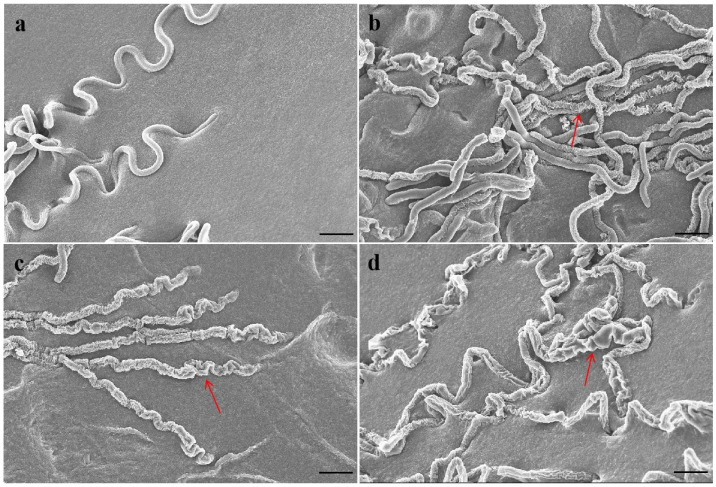
Effect of antofine on the mycelial morphology of *P. capsici*. (**a**) Not treated with antofine; (**b**) treated with antofine at EC_30_; (**c**) treated with antofine at EC_50_; (**d**) treated with antofine at EC_70_. The red arrow showed that the mycelium of *P. capsici* had obvious depression and serious wrinkles. Bar = 10 μm.

The mycelium of *P. capsici* was smooth and plump without antofine (Figure 1a). The hyphae were slightly rough and wrinkled following treatment with antofine (EC_30_), with respect to the control (Figure 1b). Compared to the control, the hyphae were rough, less full, and had obvious depression, an irregular protrusion structure, and serious wrinkles following treatment with antofine (EC_50_ and EC_70_; Figure 1c,d).

### 2.3. Effect of Antofine on the Physiological and Biochemical Characteristics of P. capsici

**Figure 2:**
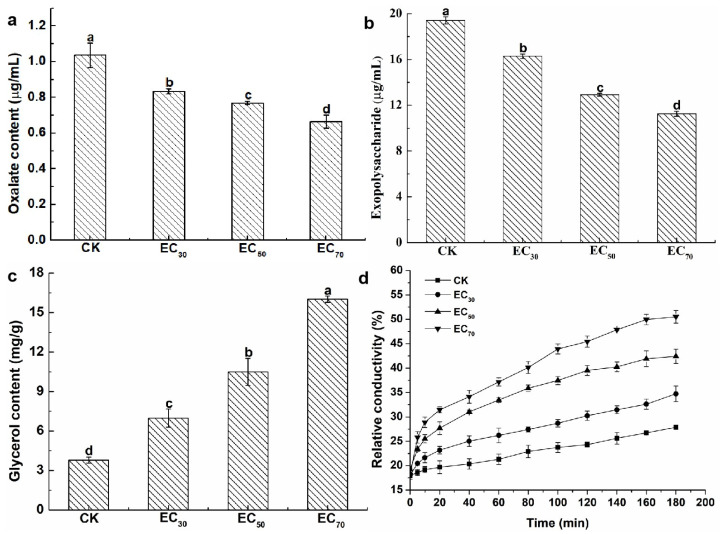
Effect of antofine on the physiological and biochemical characteristics of *P. capsici*. (**a**) Oxalate content; (**b**) exopolysaccharide content; (**c**) glycerol content; (**d**) conductivity values. Treatments: control (not treated with antofine; CK) and antofine (EC_30_, EC_50_, and EC_70_). Data represent the mean of three independent experiments. Bars represent the standard error of the experiments. Different lowercase letters indicate a significant difference (*p* < 0.05), according to Duncan’s multiple range test.

The oxalic acid content was calculated using the standard curves (Figure S1a). The oxalic acid content was 1.04 mg/mL without antofine treatment. Compared to the control, the oxalic acid content was significantly reduced by 20.19%, 25.96%, and 35.58%, respectively, following treatment with antofine at EC_30_, EC_50_, and EC_70_ (Figure 2a). The exopolysaccharide content was calculated using the standard curves (Figure S1b). The exopolysaccharide content was 19.42 μg/mL without antofine treatment. Compared to the control, the exopolysaccharide content was significantly reduced by 16.17%, 33.42%, and 42.07%, respectively, following treatment with antofine at EC_30_, EC_50_, and EC_70_ (Figure 2b). The glycerol content was calculated using the standard curves (Figure S1c). The glycerol content was 3.79 mg/g without antofine treatment. Compared to the control, the glycerol content was significantly increased by 84.17%, 176.78%, and 322.69%, respectively, following treatment with antofine at EC_30_, EC_50_, and EC_70_ (Figure 2c). The results indicate that the relative conductivity of the mycelium from *P. capsici* increased over time after treatment with different concentrations of antofine (EC_30_, EC_50_, and EC_70_). The relative conductivity increased with higher concentrations of antofine, reaching its highest relative conductivity following treatment with antofine at EC_70_ (Figure 2d).

### 2.4. Effect of Antofine on the Intracellular ROS Contents of P. capsici

**Figure 3:**
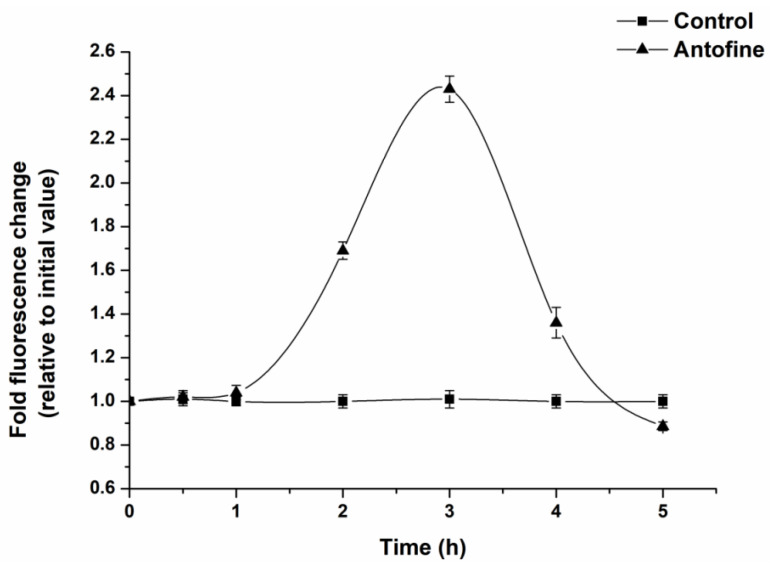
Effect of antofine on the intracellular ROS of *P. capsici*. The mycelia of *P. capsici* were treated with antofine at EC_50_, while treatment without antofine was taken as the control. Data represent the mean of three independent experiments. Bars represent the standard error of the experiments.

DCFH-DA was used as a probe to determine the intracellular ROS production of *P. capsici* within 6 h of incubation of antofine. The results show that the production of ROS in the cells of *P. capsici* exhibited a trend of first increasing and then decreasing as the incubation time increased (Figure 3). After 3 h of incubation, the ROS in the antofine treatment group reached the maximum value, and the fluorescence ratio was 2.43 times compared to the control group.

### 2.5. Effect of Antofine on the Mitochondrial Respiratory Chain Complexes of P. capsici

**Figure 4:**
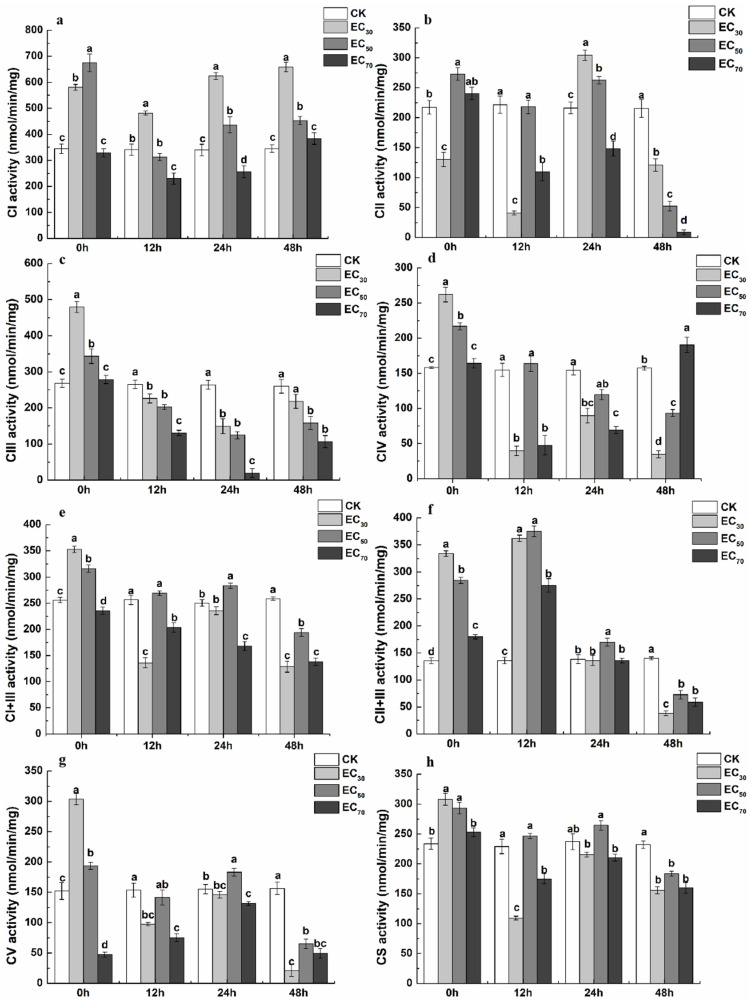
Effect of antofine on *P. capsici* mitochondrial respiratory chain complexes. (**a**) Complex I (CI); (**b**) complex II (CII); (**c**) complex III (CIII); (**d**) complex IV (CIV); (**e**) complex I + III (CI + III); (**f**) complex II + III (CII + III); (**g**) complex V (CV); (**h**) citrate synthase (CS). The mycelia of *P. capsici* were treated for 0, 12, 24, and 48 h with antofine (EC_30_, EC_50_, and EC_70_) or without antofine (CK). Data represent the mean of three independent experiments. Different lowercase letters indicate a significant difference (*p* < 0.05) according to Duncan’s multiple range test.

Compared to the control, the activity of complex I was significantly increased following treatment with antofine at EC_30_ and EC_50_ for 0 h (68.38% and 95.94%, respectively). Compared to the control, the activity of complex I was significantly increased by 41.30% when treated with antofine at EC_30_ and significantly decreased by 32.51% when treated with antofine EC_70_ after 12 h of treatment. Compared to the control, the complex I activity significantly increased by 83.55% and 28.29% when treated with antofine at EC_30_ and EC_50_ and decreased significantly by 24.60% when treated with antofine at EC_70_ after 24 h of treatment. Compared to the control, the complex I activity significantly increased by 90.46% and 31.07% when treated with antofine at EC_30_ and EC_50_, while there was no significant difference between antofine at EC_70_ and the control after 48 h of treatment (Figure 4a). Compared to the control, the complex II activity decreased significantly by 40.09% after exposure to antofine at EC_30_ and increased significantly by 25.57% after treatment with antofine at EC_50_, while there was no significant difference between the control and antofine at EC_70_ following 0 h of treatment. Compared to the control, the complex II activity was significantly increased by 40.83% and 21.49% when treated with antofine at EC_30_ and EC_50_ for 24 h and decreased significantly by 31.34% when treated with antofine at EC_70_. The complex II activity was significantly decreased following treatment with antofine at EC_30_, EC_50_, and EC_70_ (43–96% compared with the control) for 48 h (Figure 4b). Compared to the control, the complex III activity was increased significantly following treatment with antofine at EC_30_ and EC_50_ for 0 h (78.78% and 27.88%, respectively). Compared to the control, the complex III activity was significantly decreased by 43.35%, 52.88%, and 92.52%, respectively, after treatment with antofine at EC_30_, EC_50_, and EC_70_ for 24 h (Figure 4c). Compared to the control, the complex IV activity was increased significantly following treatment with antofine at EC_30_ and EC_50_ for 0 h (65.59% and 37.17%, respectively). Compared to the control, the complex IV activity was significantly inhibited by 42–74% when treated with antofine at EC_30_ and EC_70_, with no significant differences between the control and antofine at EC_50_ for 12 and 24 h. The complex IV activity was significantly decreased following treatment with antofine at EC_30_ and EC_50_ (40–78% compared to the control) and increased significantly following treatment with antofine at EC_70_ (20.95% compared to the control) for 48 h (Figure 4d). Compared to the control, the complex I + III activity increased significantly following treatment with antofine at EC_30_ and EC_50_ for 0 h (37.65% and 23.52%, respectively) and significantly decreased by 8.04% following treatment with antofine at EC_70_. Compared to the control, the complex I + III activity was inhibited by 20–47% when treated with antofine at EC_30_ and EC_70_ for 12 h, with no significant differences between the control and antofine at EC_50_. The activity of complex I + III was significantly increased by 13.27% following treatment with antofine at EC_50_ for 24 h, while it was significantly decreased following treatment with antofine at EC_30_, EC_50_, and EC_70_ (25–50% compared with the control) for 48 h (Figure 4e). Compared to the control, the complex II + III activity was significantly increased by 33–176% following treatment with antofine at EC_30_, EC_50_, and EC_70_ for 0 and 12 h. The complex II + III activity was significantly increased by 22.85% (compared to the control) following treatment with antofine at EC_50_ for 24 h, while there was no significant difference between the control and antofine at EC_30_ and EC_70_. The complex II + III activity was significantly decreased (48–73% compared to the control) following treatment with antofine at EC_30_, EC_50_, and EC_70_ for 48 h (Figure 4f). Compared to the control, the complex V activity was significantly increased by 99.49% and 27.21%, respectively, following treatment with antofine at EC_30_ and EC_50_ for 0 h. The complex V activity was significantly decreased by 15–69% (compared to the control) following treatment with antofine at EC_70_ for 0, 12, and 24 h, and it was significantly decreased (58–87% compared to the control) following treatment with antofine at EC_30_, EC_50_, and EC_70_ for 48 h (Figure 4g). Compared to the control, the citrate synthase activity was significantly increased following treatment with antofine at EC_30_ and EC_50_ for 0 h (31.93% and 25.56%, respectively), and there was no significant difference between the control and antofine at EC_70_. The activity of citrate synthase was significantly decreased (52.24% and 23.61%, respectively, compared to the control) following treatment with antofine at EC_30_ and EC_70_ for 12 h, and there was no significant difference between the control and antofine at EC_50_. The activity of citrate synthase was significantly decreased (20–33% compared to the control) following treatment with antofine at EC_30_, EC_50_, and EC_70_ for 48 h (Figure 4h).

## 3. Discussion

Natural products have become the main direction for crop disease control and represent one of the technologies for achieving sustainable development and ensuring the safety of food production. China attaches great importance to the development of biopesticide technology products, which are designated as a strategic initiative for the country’s high-quality and green development [19]. One of the important ways to create new biopesticides is to study natural chemical products, especially active plant secondary metabolites, followed by lead optimization to synthesize candidate compounds. More importantly, the mechanism of action of active compounds can be studied, and new targets can be explored to provide a theoretical basis for the research and development of plant-derived pesticides [20].

In our continued efforts to screen fungicidal substances from toxic psammophytes, we discovered antofine, an alkaloid that has a good antifungal effect against a variety of pathogens. Previously published research has suggested that antofine exhibits profound antiproliferative activities in a variety of cancer cells [21]. The present study verified that antofine has an obvious inhibitory effect against *P. capsici*, with an EC_50_ value of 5.0795 μg/mL (Table 1). Meanwhile, antofine has a significant inhibitory effect on hyphal growth, with the hyphae becoming rough and less full with obvious depression, an irregular protrusion structure, and serious wrinkles (Figure 1). The results are in agreement with previous studies, certifying that antofine has a good antifungal effect [22,23,24]. Oxalic acid is the smallest and most acidic binary carboxylic acid in nature, and it is widely distributed in plants, animals, and fungi [25]. The oxalic acid secreted by pathogenic fungi is an important pathogenic factor in the interaction between pathogenic fungi and plants. Oxalic acid acidifies plant cells, also enhancing the activity of pathogenic cell wall degrading enzymes; disrupts the burst of active oxygen species in plant cells; chelates calcium ions; and affects the normal operation of the calcium ion signaling pathway. Oxalic acid can directly poison plant cells and cause electrolyte loss of plant cells. In the early stage of infection, oxalic acid inhibits the autophagy of plant cells, while in the later stage, it promotes the apoptosis of plant cells [26]. It has been found that the yield of oxalic acid is positively correlated with pathogenicity [27], which is consistent with the present study, in which the oxalic acid of *P. capsici* decreased significantly (20–36% compared to the control) as the antofine concentration increased (Figure 2a). Exopolysaccharides are pathogenic factors of many bacteria that block the vascular bundle of host plants, rupture the catheter, and promote the colonization and diffusion of pathogenic bacteria [28,29]. Previous results have shown that the exopolysaccharide content in *Pseudopestalotiopsis camelliae-sinensis* decreases significantly as the phenazine-1-carboxylic acid concentration increases [30], which is consistent with the present study, in which the exopolysaccharides of *P. capsici* decreased significantly (16–42% compared to the control) as the antofine concentration increased (Figure 2b). Glycerol is the main factor of osmoregulation by microorganisms [31,32]. After antofine treatment, the glycerol content significantly increased by 84–322.69% compared to the control (Figure 2c). The conductivity is related to the concentration of ions in the solution [33]. This study showed that the relative conductivity of *P. capsici* increased significantly after treatment with increasing antofine concentrations (Figure 2d), indicating that the intracellular electrolytes of *P. capsici* were discharged and the membrane permeability increased, which is consistent with previous studies [34,35]. Therefore, after antofine treatment, the glycerol content and relative conductivity of *P. capsici* were significantly increased, causing a large influx of external water and resulting in cell expansion, rupture, and death. These results have important guiding significance for exploring the mechanism of action of antofine against *P. capsici*.

ROS is a general term for oxygen-containing free radicals and peroxides that easily form free radicals, including O_2_^−^, H_2_O_2_, OH^−^, O_3_, and ^1^O_2_. ROS are mainly produced during the transmission process of the respiratory chain in the inner mitochondrial membrane. This study showed that the ROS in the antofine treatment group reached the maximum value, and the fluorescence ratio was 2.43 times compared to the control group after 3 h of incubation (Figure 3). The results showed that antofine may damage the mitochondrial structure of *P. capsici* or affect the pathways related to energy metabolism, and then affect the mitochondrial respiratory chain, resulting in the burst of ROS. Research has shown that sanguinarine is a phenindolicidine alkaloid, which could lead to apoptosis and a decrease in the mitochondrial membrane potential [36], which is similar to the findings of this study. The intracellular ATP content of the *Penicillium digitatum* Pds01 strain decreases when treated with antofine [17], which suggests that antofine may affect mitochondrial function and is consistent with this study. In this study, the activity of the respiratory chain complexes of *P. capsici* increased (23–147%), except for the complex II, when treated with antofine at EC_30_ and EC_50_ for 0 h. Meanwhile, the activity of the respiratory chain complexes was significantly inhibited, except for complex I and complex IV when treated with antofine for 48 h. However, antofine treatment for 12 and 24 h influenced the activity of complex III in a dose- and time-dependent manner (Figure 4). Therefore, we speculate that the mitochondrial respiratory chain complex enzyme of *P. capsici* is not the target of antofine. The mitochondrion is a two-layer membrane-wrapped organelle for material metabolism and energy synthesis, which is the central hub controlling cell life activities [37]. The pyruvate, produced by glycolysis, enters the mitochondrial matrix, passes through the tricarboxylic acid cycle, forms reduced nicotinamide adenine dinucleotide (NADH) and reduces flavin adenosine dinucleotide (FADH_2_) into the respiratory chain and completely releases ATP to form CO_2_ and H_2_O [38]. There are two electron transport pathways in the mitochondrial electron transport chain, and complex III is essential in both respiratory pathways [39]. The pathway related to energy metabolism is inhibited, which may also lead to decreased mitochondrial complex activity.

## 4. Materials and Methods

### 4.1. Chemicals and Pathogen

Antofine (active ingredient > 90%) was provided by the Plant Protection Laboratory, College of Life Sciences, Yulin University, China. A Reactive Oxygen Species (ROS) kit was purchased from Beijing Solarbio Science and Technology Co., Ltd. (Beijing, China). Cytochrome c from bovine heart, rotenone, oligomycin, and antimycin A were used in the determination of mitochondrial respiratory chain complex activity and were purchased from Shanghai Yuanye Bio-Technology Co., Ltd. (Shanghai, China). V_8_ juice was purchased from the Campbell Soup Company (Camden, NJ, USA). The other chemicals and reagents of analytical grade were purchased from Shanghai Yuanye Bio-Technology Co., Ltd. (Shanghai, China).

The strain of *P. capsici* was collected from pepper fields in Yulin City, Shaanxi Province, China, in 2020. The strain was provided by the Plant Protection Laboratory, College of Life Sciences, Yulin University, China. The sampling fields were exposed to small amounts of pesticides. The strain of *P. capsici* was identified by colony morphology and sequence analysis and stored in 10% V_8_ agar culture medium at 4 °C.

The strain was grown on 10% V_8_ agar culture medium at 25 °C for 5 days before use. The mycelia of *P. capsici* were produced in 10% V_8_ fluid medium (without agar) on a rotary shaker (175 rev/min) at 25 °C. After 5 days of growth, the cultures were filtered through a sterile cheesecloth to collect the mycelia. Then, a 10% V_8_ agar culture medium was prepared with 100 mL of V_8_ juice, 15 g of agar, 0.2 g of CaCO_3_, and 900 mL of distilled water.

### 4.2. Determination of the Effect of Antofine on the Mycelial Growth of P. capsici

Mycelia plugs (5 mm in diameter) from the leading edge of *P. capsici* were transferred to a series of 10% V_8_ agar culture medium plates containing 0, 2.5, 5, 10, 20, or 40 μg/mL of antofine. When the control colony diameter exceeded 2/3 of the plate diameter, the crossing method was used to measure the colony diameter from two vertical directions, and the 5 mm diameter of the mycelia plugs was deducted. The inhibition rate of mycelial growth was carried out according to the method described by Wang et al. [40]. All tests were conducted three times, and each experiment was repeated thrice. The effective concentrations (EC_30_, EC_50_, and EC_70_) of antofine against *P. capsici* were assessed based on a linear regression of the colony diameter versus the log-transformed fungicide concentration.

### 4.3. Observation of the Effect of Antofine on the Mycelial Morphology of P. capsici

The mycelium plugs were transferred to 10% V_8_ agar culture medium plates containing antofine (EC_30_, EC_50_, and EC_70_) and without antofine (control). The sample preparation was slightly modified according to Cleary et al.’s method [41]. After 3 days at 25 °C, the colony edge (2 × 2 mm) of *P. capsici* was taken with a scalpel, totaling five samples for each treatment. The samples were quickly fixed in 2.5% glutaraldehyde and vacuumed in a needle tube to ensure that the samples sank adequately. The samples were rinsed three times with phosphoric acid buffer (pH 7.2) for 15 min each time, and 30%, 50%, 70%, 80%, and 90% ethanol was dehydrated once each, while 100% ethanol was dehydrated three times. The samples were freeze-dried overnight and observed by scanning electron microscopy (Hitachi, Tokyo, Japan) after gold spraying.

### 4.4. Determination of the Effect of Antofine on the Physiological and Biochemical Characteristics of P. capsici

#### 4.4.1. Determination of the Effect of Antofine on the Oxalic Acid Content of *P. capsici*

The mycelium plugs were transferred to a triangular flask containing 100 mL of 10% V_8_ fluid medium and incubated on a rotary shaker (175 rev/min) at 25 °C for 3 days. Antofine (EC_30_, EC_50_, and EC_70_) was then added to a triangular flask (without antofine as the control), and the supernatant was collected by centrifugation after 24 h. The determination of the oxalic acid content was slightly modified according to the method of Duan et al. [42]. First, 4 mL of a FeCl_3_ solution (0.5 mg/mL), 40 mL of HCl-KCl buffer (KCl 50 mM, pH 2), and 2.4 mL of sulfosalicylic acid (5 mg/mL) were added to a 50 mL volumetric flask. Then, 0, 0.1, 0.2, 0.4, 0.8, and 1.6 mL of a sodium oxalate standard solution (2 mg/mL) were added successively, and the volume was fixed with distilled water to 50 mL. After mixing and incubating at 25 °C for 30 min, the absorbance was measured using a spectrophotometer (752N PLUS, INESA, Shanghai, China) at 510 nm. A standard curve was drawn based on the absorption value and sodium oxalate. The absorbance of the supernatant was determined using a spectrophotometer at 510 nm. The oxalic acid content in *P. capsici* treated with antofine was calculated according to the standard curve. All tests were conducted three times, and each experiment was repeated thrice.

#### 4.4.2. Determination of the Effect of Antofine on the Exopolysaccharide Content of *P. capsici*

The determination of the exopolysaccharide content was slightly modified according to Rao and Pattabiraman’s method [43]. The glucose was dissolved in sterile water with the following standard solution amounts: 0, 30, 60, 90, 120, 150, 180, and 210 μg/mL. Then, 2 mL of a glucose solution and 1 mL of a 5% phenol solution were added to a 10 mL centrifuge tube. Next, 5 mL of concentrated sulfuric acid was slowly added along the tube wall, mixed, and incubated at 25 °C for 30 min. The absorbance of the solution was measured using a spectrophotometer at 490 nm. A standard curve was drawn based on the absorption value and glucose. The culture of *P. capsici* was consistent with Section 4.4.1. The culture medium was centrifuged at 12,000 rpm for 15 min and the supernatant was taken, precipitated with anhydrous ethanol, and dried at 50 °C. The precipitation was dissolved in 5 mL of sterile water, and the absorbance was determined at 490 nm. The exopolysaccharide content was calculated according to the standard curve.

#### 4.4.3. Determination of the Effect of Antofine on the Glycerol Content of *P. capsici*

The determination of the glycerol content was slightly modified according to the method of Duan et al. [42]. Glycerol was prepared with distilled water into different standard solution concentrations: 0, 0.0025, 0.003, 0.004, 0.005, 0.006, 0.008, and 0.01 g/mL. First, 5 mL of a glycerol standard solution was added into a 10 mL centrifuge tube, and then 0.5 mL of a 0.05 g/L CuSO_4_ solution and 1.75 mL of a 0.05 g/mL NaOH solution were added. The absorbance was measured at 630 nm using a spectrophotometer after mixing and filtering. A standard curve was drawn based on the absorbance and glycerol. The culture of *P. capsici* was consistent with Section 4.4.1. Next, 0.5 g of the mycelium was added into 20 mL of sterile water for grinding (with quartz sand), and the milled mixture was transferred into a 50 mL centrifuge tube and heated at 80 °C for 15 min in water. The milled mixture was centrifuged at 9000 rpm for 7 min, and the supernatant was taken. The absorbance of the supernatant was determined using a spectrophotometer at 630 nm. The glycerol content in *P. capsici* treated with antofine was calculated according to the standard curve.

#### 4.4.4. Determination of the Effect of Antofine on the Cell Membrane Permeability of *P. capsici*

The culture of *P. capsici* was consistent with Section 4.4.1. First, 0.3 g of the mycelium was suspended in 10 mL of sterile water. The conductivity was determined using a conductivity meter (DDBJ-350, Hangzhou Qiwei Instrument Co., Ltd., Hangzhou, China) after 0, 5, 10, 20, 40, 60, 80, 100, 120, 140, 160, and 180 min. After 180 min, the mycelium solution was boiled for 5 min, and the final conductivity was measured after the solution was cooled to room temperature. The relative conductivity was calculated according to the method of Duan et al. [42].

### 4.5. Determination of the Effect of Antofine on the Intracellular ROS Content of P. capsici

The ROS levels were determined according to the instructions of the DCFH-DA Kit (Solarbio, Beijing, China). The culture of *P. capsici* was consistent with Section 4.4.1. The mycelium was weighed to 70 mg and washed 3 times with ultra-pure water. The mycelium was incubated with 5.0795 μg/mL of antofine for 30 min, 1 h, 2 h, 3 h, 4 h, and 5 h, respectively, while that without antofine was taken as the control. DCFH-DA was diluted to 10 μmol/L with phosphate buffer (pH 7.2). The incubated mycelium was suspended in diluted DCFH-DA and incubated at 37 °C for 20 min. The incubated mycelium was washed with phosphate buffer (pH 7.2) 3 times, then ultrasonicated in an ice bath for 3 min. The ultrasonicated mycelium was centrifuged at 4 °C at 25,000× *g* for 5 min. The absorbance of the supernatant was determined under an excitation light of 488 nm and an emission light of 525 nm on a fluorescence spectrophotometer (Hitachi).

### 4.6. Determination of the Effect of Antofine on the Mitochondrial Respiratory Chain Complexes of P. capsici

The culture of *P. capsici* was consistent with Section 4.4.1. Antofine (EC_30_, EC_50_ and EC_70_) was then added to a triangular flask (without antofine as control), and the mycelia were collected at 0, 12, 24, and 48 h. The mitochondrial respiratory chain complexes were prepared according to Tamura et al. [44]. The activity of the respiratory chain complexes was assessed using an ultraviolet–visible spectrophotometer 752N PLUS (INESA, Shanghai, China). The activity of complexes I, II, III, IV, V I + III, and II + III and citrate synthase (CS) was measured according to Wang et al. [40].

### 4.7. Data Analysis

Statistical analysis was performed using SPSS software (v19.0, IBM, Chicago, IL, USA). The significance of the difference between treatments was determined using Duncan’s multiple-range test. *p* < 0.05 was considered statistically significant.

## 5. Conclusions

In summary, the research revealed that antofine has a high suppressive effect on the mycelial growth of *P. capsici*. Antofine may present a good opportunity to control *P. capsici* by changing the membrane permeability, increasing glycerol biosynthesis, and decreasing oxalic acid and exopolysaccharides secretion. More importantly, antofine treatment could induce the burst state of ROS, which has a certain inhibitory effect on the respiratory chain complexes, indicating that antofine is likely to affect the pathways related to the energy metabolism of *P. capsici*. To the best of our knowledge, this is the first report on the mechanism of antofine against *P. capsici*, although further research is needed to clarify the target of antofine.

## Figures and Tables

**Table 1:** The effect of antofine on the mycelial growth of *P. capsici*.

Concentration of Antofine (μg/mL)	Growth Inhibition Rate (%)	Regression Equation	EC_30_ (μg/mL)	EC_50_ (μg/mL)	EC_70_ (μg/mL)	r
2.5	32.61 ± 1.06 e	Y = 4.4047 + 0.8844x	1.2891 (0.5852–2.8277) ^1^	5.0795 (3.8846–7.4818)	19.0233 (14.9513–25.5944)	0.9778
5	57.78 ± 1.32 d					
10	63.89 ± 1.29 c					
20	70.00 ± 1.44 b					
40	87.22 ± 1.67 a					

^1^ Data represent the 95% confidence limit; data represent the mean value of triplication, with different lower case letters indicating a significant difference (*p* < 0.05). The EC_30_, EC_50_, and EC_70_ values were assessed based on log-transformation analysis by the data processing system.

## Data Availability

All data generated or analyzed during this study are included in this published article.

## Supplementary Materials

The following supporting information can be downloaded at https://www.mdpi.com/article/10.3390/molecules29091965/s1, Figure S1: Standard curve for determination of oxalate, exopolysaccharide and glycerol content.
