# Supplementary material for: The Effects of Antofine on the Morphological and Physiological Characteristics of Phytophthora capsici
Source: Molecules. 2024 Apr 25;29(9):1965. doi: 10.3390/molecules29091965 (PMC11085548; doi:10.3390/molecules29091965)
Supplement: Supplementary file 1 [file molecules-29-01965-s001.zip › molecules-2911722-supplementary.pdf]

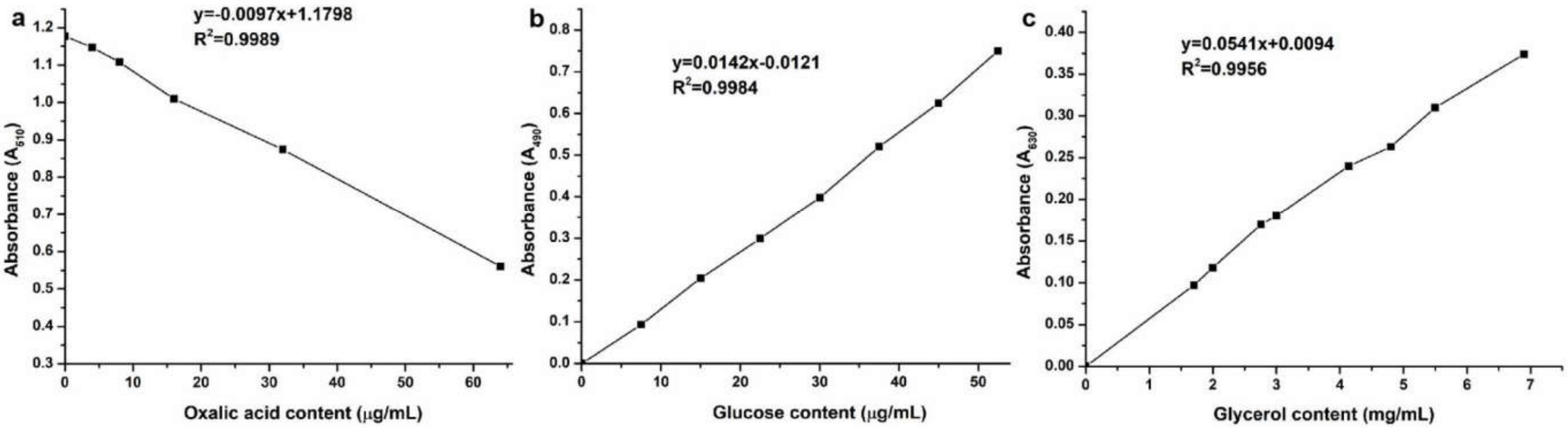

Figure S1. Standard curve. (a) Standard curve for determination of oxalate content; (b) standard curve for determination of exopolysaccharide content; (c) standard curve for determination of glycerol content
